# Supplementary material for: Enhancing Methane Recovery with Cryogenic Liquid CO2 Cyclic Injection: Determination of Cyclic Injection Parameters
Source: Int J Environ Res Public Health. 2022 Oct 13;19(20):13155. doi: 10.3390/ijerph192013155 (PMC9603266; doi:10.3390/ijerph192013155)
Supplement: Supplementary file 1 [file ijerph-19-13155-s001.zip › ijerph-1906502-supplementary.pdf]

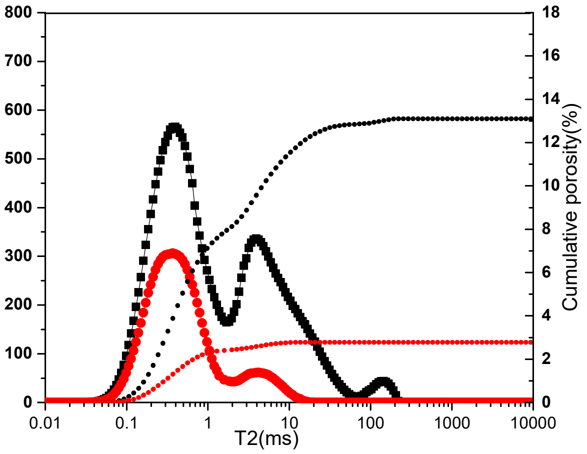

N_c=0

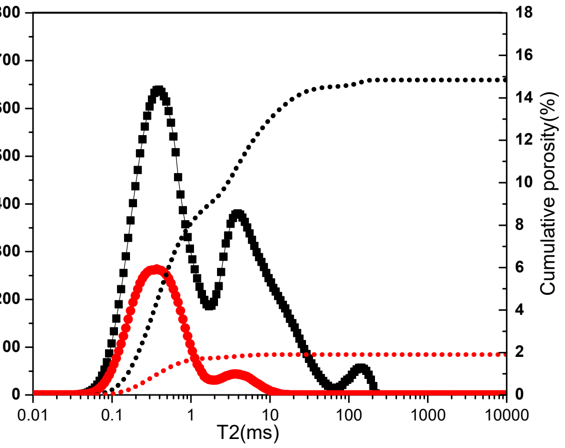

N_c =2

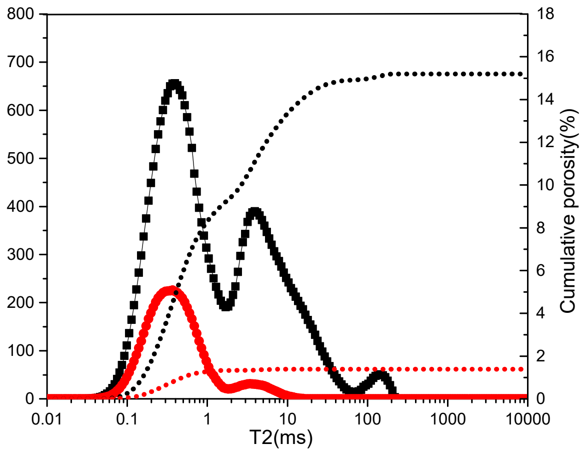

N_c =3

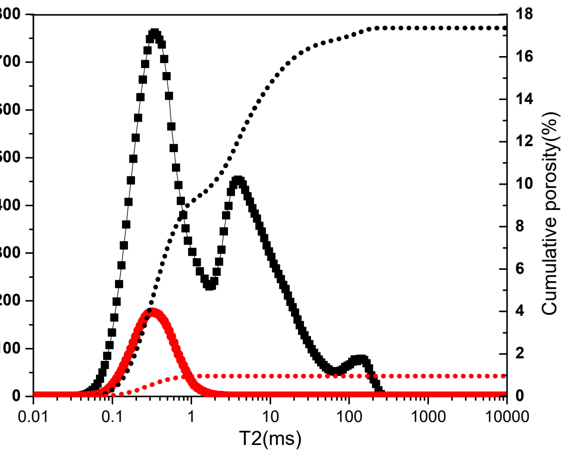

N_c =4

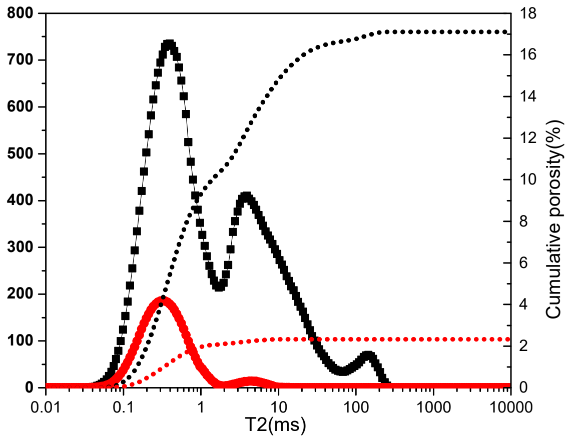

N_c=8

Figure S1. Schematic diagram of porosity percentage and cumulative porosity of lignite at water-saturated and irreducible water conditions

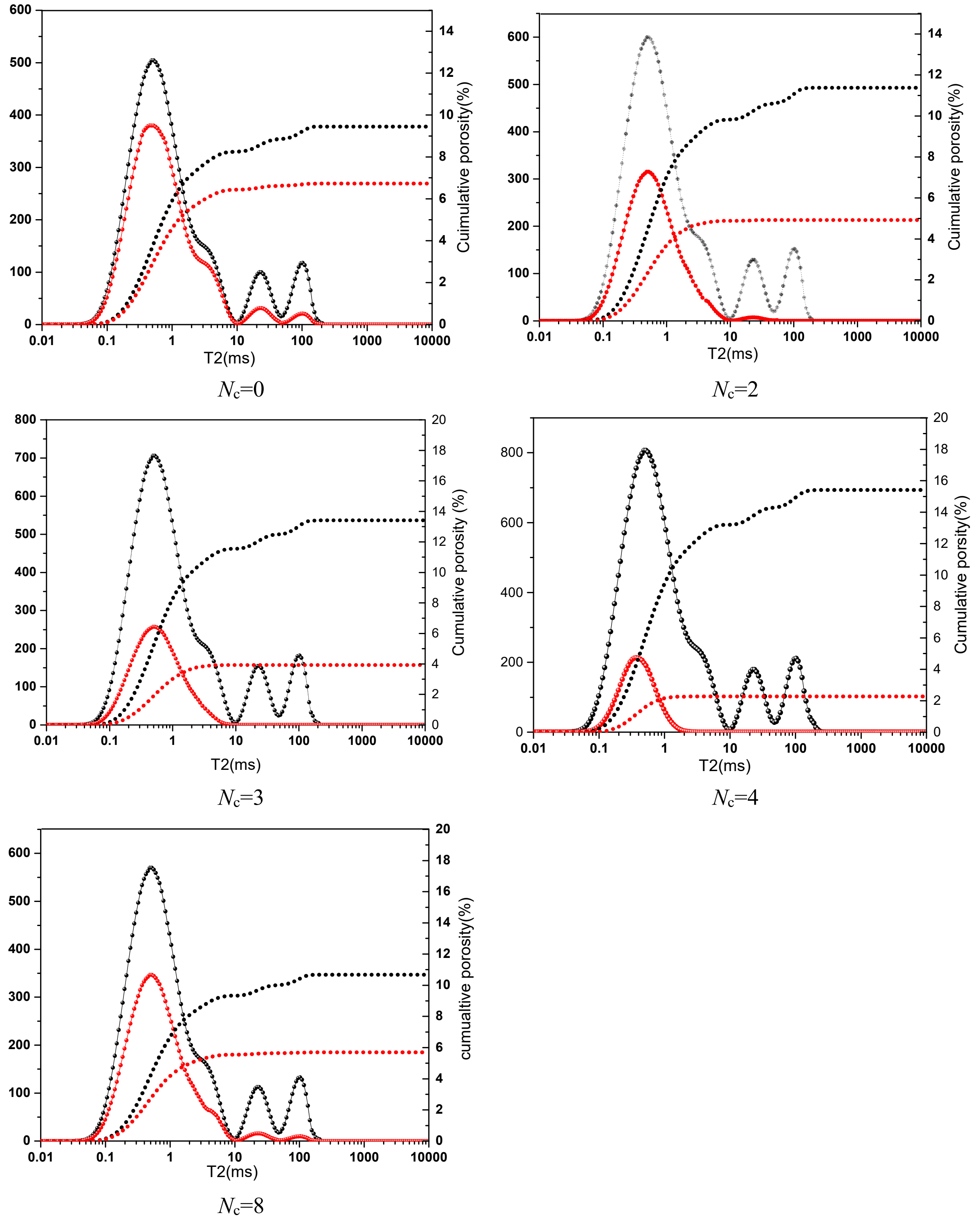

Figure S2. Schematic diagram of porosity percentage and cumulative porosity of gas coal at water-saturated and irreducible water conditions

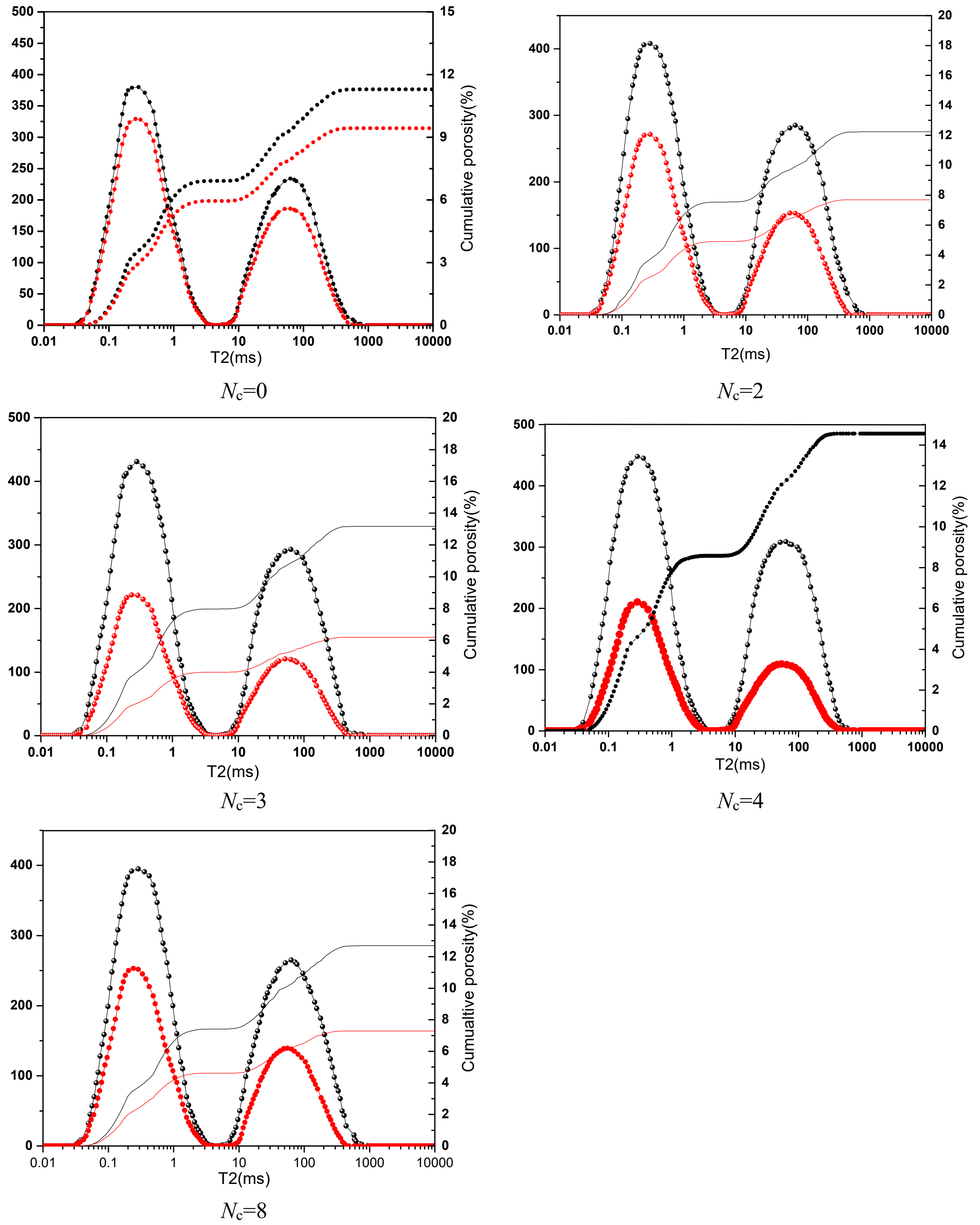

Figure S3. Schematic diagram of porosity percentage and cumulative porosity of 1/3 coking coal at water-saturated and irreducible water conditions
